# Effects of High Voltage Atmospheric Cold Plasma Treatment on the Number of Microorganisms and the Quality of *Trachinotus ovatus* during Refrigerator Storage

**DOI:** 10.3390/foods11172706

**Published:** 2022-09-05

**Authors:** Zhicheng Cai, Jiamei Wang, Chencheng Liu, Gu Chen, Xiaohan Sang, Jianhao Zhang

**Affiliations:** 1 College of Food Science and Engineering, Hainan University, Haikou 570228, China; 2 College of Food Science and Technology, Nanjing Agricultural University, Nanjing 210095, China

**Keywords:** high voltage atmospheric cold plasma, *Trachinotus ovatus*, number of microorganisms, quality, refrigeration

## Abstract

In order to investigate the effects of high voltage atmospheric cold plasma (HVACP) treatment on the number of microorganisms in and the quality of *Trachinotus ovatus* during refrigerator storage, fresh fish was packaged with gases CO_2_:O_2_:N_2_ (80%:10%:10%) and treated by HVACP at 75 kV for 3 min; then, the samples were stored at 4 ± 1 °C for nine days. The microbial numbers, water content, color value, texture, pH value, thiobarbituric acid reactive substance (TBARS), and total volatile base nitrogen (TVB-N) values of the fish were analyzed during storage. The results showed the growth of the total viable bacteria (TVB), psychrophilic bacteria, *Pseudomonas* spp., H_2_S-producing bacteria, yeast, and lactic acid bacteria in the treated samples was limited, and they were 1.11, 1.01, 1.04, 1.13, 0.77, and 0.80 log CFU/g^−1^ lower than those in the control group after nine days of storage, respectively. The hardness, springiness, and chewiness of the treated fish decreased slowly as the storage time extended, and no significant changes in either pH or water content were found. The lightness (L*) value increased and the yellowness (b*) value decreased after treatment, while no changes in the redness (a*) value were found. The TBARS and TVB-N of the treated samples increased to 0.79 mg/kg and 21.99 mg/100 g, respectively, after nine days of refrigerator storage. In conclusion, HVACP can limit the growth of the main microorganisms in fish samples effectively during nine days of refrigerator storage with no significant negative impact on their quality. Therefore, HVACP is a useful nonthermal technology to extend the refrigerator shelf-life of *Trachinotus ovatus*.

## 1. Introduction

The *Trachinotus ovatus* belongs to the class of bony fish and the genus *Pompano*, also known as Pompano and silverfish. It is an important fish for the marine aquaculture economy in Asia and the Pacific region; it contains a variety of amino acids necessary for the human body [1], and its nutritional ratio reaches the WHO/FAO standard [2]. It is rich in polyunsaturated fatty acids (PUFA), such as docosahexaenoic acid (DHA) and eicosapentaenoic acid (EPA), and the fish is palatable, nutritious, and popular with consumers [3,4]. It is estimated that the output of cage aquaculture will reach 38,900 tons, and the economic value will reach nearly CNY 1.1 billion in 2022 in China [5].

*T. ovatus* is susceptible to microbial contamination during processing and transportation. In addition, degradation of endogenous enzymes in fish can lead to spoilage. At present, the fish is sold as fresh, frozen, or refrigerated, all of which have a short shelf life, which reduces the economic value of golden pompano products [6]. The fresh-keeping technologies for *T. ovatus* are mainly freezing, modified atmosphere packaging (MAP) [7], and film coating [8].

At present, packaging technologies combined with cold chain storage are mainly used for aquatic product fresh-keeping. Zhang et al. [7] studied the quality changes in *T. ovatus* packaged with modified atmosphere packaging during refrigerator storage and found that MAP could effectively inhibit the increase in the pH and TVB-N values during storage, and the shelf life was 4–5 days longer than that of the air packaging group.

Plasma, a partially or fully ionized gas, is considered the fourth state of matter, with solid, liquid, and gas as the other three. It contains two main kinds of reactive oxygen and nitrogen species (RONS), which including long shelf-life species (O_3_, H_2_O_2_, NO_2_^−^, NO_3_^−^) and short shelf-life species (·OH, HOO, O_2_^−^, ONOO−) [9]. RONS can attack and damage the peptidoglycan bond structure of cell walls (C-O, C-C, C-N), resulting in the leakage of DNA, nucleic acid, and other substances in the cytoplasm to cause the death of cells [10]. It has been shown that cold plasma can inhibit the growth of bacteria in certain kinds of food products [11,12,13,14].

In recent years, studies on cold plasma application in aquatic products have become more prevalent and have focused primarily on the quality changes during storage after treatment. Mohamed et al. [15] studied the effects of dielectric barrier discharge (DBD) cold plasma voltages and treatment time on the quality of tilapia during refrigerator storage and found that the cold plasma treatment had a significant effect on the enzyme activities and the number of microorganisms; the shelf-life was extended to 10 days when samples were treated at 60 kV for 4 min. Albertos et al. [16] investigated the effect of atmospheric cold plasma on the microbial and quality parameters of mackerel fillets treated with different voltages and times. Puligundla et al. [17] studied the decontamination effect of *Gwamegi* with a corona discharge plasma jet (CDPJ) and reported that the CDPJ treatment inhibited the growth of bacteria effectively with no significant changes found in the physicochemical properties. Zouelm et al. [18] studied the effects of cold plasma application on the quality and chemical spoilage of pacific white shrimp during refrigerated storage and concluded that CP treatment for 90 s was the most efficient condition to extend the shelf life of shrimp. Pérez-Andrés et al. [19] investigated the lipids and protein oxidation of mackerel after cold plasma treatment and found no significant changes in lipid oxidation, and the formation of carbonyls was accelerated at the end of the storage. Therefore, cold plasma treatment is potentially suitable as a processing technology in the aquatic industry. So far, few reports on the effects of cold plasma treatment on *T. ovatus* have been published. In order to investigate the effects of high-voltage atmospheric cold plasma (HVACP) treatment on *T. ovatus* during refrigerator storage, the amount of microorganisms, thiobarbituric acid reactive substance (TBARS), total volatile base nitrogen (TVB-N), texture, pH, and moisture content of fish during refrigeration were analyzed in this study.

## 2. Materials and Methods

### 2.1. Fish Sample Preparation

Freshly slaughtered *T. ovatus* (weight 1000 ± 50 g) were purchased from a local market in Haikou, China. The fish were transported to the laboratory within 1 h in an ice-filled cooler. The fish were deboned and cut into pieces (100 ± 5 g), and the fish meat was sealed in a food packaging tray with a packaging machine (MAP-H360, Senrui Preservation Equipment Co., Ltd., Suzhou, China); the gas in the tray was CO_2_:O_2_:N_2_ = 80%:10%:10%.

### 2.2. HVACP Treatment

**Figure 1:**
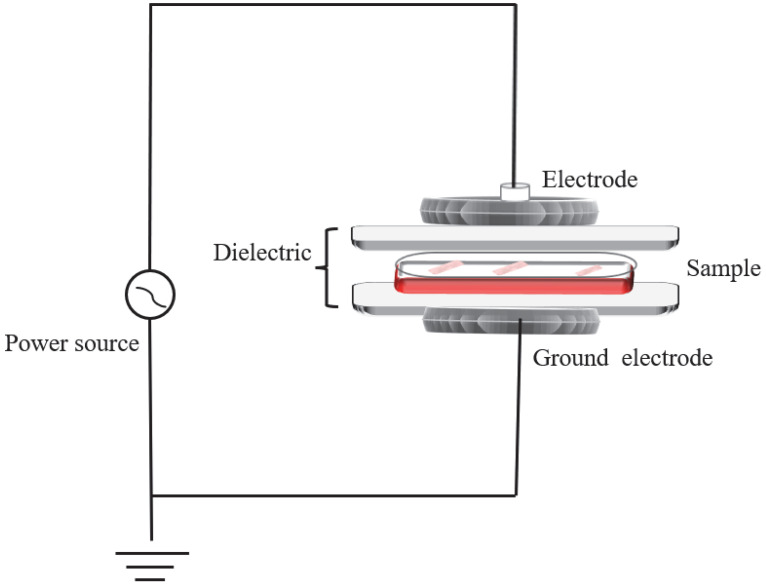
Schematic diagram of high voltage atmospheric cold plasma system.

The HVACP treatment system was the same as described in Wang et al. [20], and the schematic diagram of the system is shown in Figure 1. The sealed tray was put between the two discharges and treated directly at 75 kV for 3 min; then, it was stored in a refrigerator (4 °C) for 9 d. Samples were taken for analysis at 1, 3, 5, 7, and 9 d. The controls without cold plasma treatment were analyzed at the same times.

### 2.3. Microbiological Analyses

Microbial counts were analyzed according to the method described in Wang et al. [21] with little modification. The fish from each package (5 g) was placed in a sterile bag with 45 mL sterile 1X PBS solution, and the bag was shaken for 1 min. The solution was diluted by 1:10 for series times with sterile 1X PBS solution; then, 100 µL of each dilution was removed and spread on agar plates.

(a) The total bacteria count and psychrophilic bacteria were determined by Plate Count Agar (PAC) (Hopebio, Qingdao, China) incubated at 37 °C for 48 h and 4 °C for 10 d, respectively.
(b) *Pseudomonas* spp., were analyzed by agar plates of Pseudomonas Agar Base with added CFC (Cetrimide, Fucidine, Cephalosporine) (Hopebio, Qingdao, China) and incubated at 25 °C for 48 h.
(c) H_2_S-produing (Hydrogen sulfide-producing) bacteria were spread on iron agar plates (Hopebio, Qingdao, China) and incubated at 37 °C for 48 h.
(d) Yeast was analyzed with Potato Dextrose Agar (PDA) plates (Hopebio, Qingdao, China) and incubated at 28 °C for 5 d.
(e) Lactic acid bacteria were determined on Man Rogosa Sharpe agar (MRS Agar) (Hopebio, Qingdao, China) and incubated at 37 °C for 72 h.

The number of microbial colonies was counted and expressed as log CFU/g fish weight.

### 2.4. Water Content

A clean weighing dish was put in a drying oven at 101~105 °C and dried to a constant weight. We added about 5 ± 0.0001 g minced fish meat to the weighing dish and put it in a drying oven at 101~105 °C to dry it to a constant weight; then, we calculated the moisture content.

### 2.5. Surface Color Measurement

The brightness value (L*), red–green value (a*), and yellow–blue value (b*) of the fish meat were measured by a colorimeter (CR-10, Konica Minolta, Shenzhen, China). The manufacturer’s standard white plate was used for calibration.

### 2.6. Texture Profile Analysis (TPA)

The TPA of fish meat was carried out according to the method of Fidalgo et al. [22] with little modification. The TPA of the samples was determined using a texture analyzer (CT3, Brookfield, Waukesha, WI, USA) with a cylindrical probe (6 mm in diameter) at a test speed of 4 mm/s and a test distance of 2 mm. Three points were measured for each slice of fish, and the results were averaged.

### 2.7. pH

The fish meat (5 g) was homogenized with 45 mL saturated KCl solution for 1 min; then, the pH value of the homogenate was determined using a pH meter (REX, pHS-3C, Shanghai, China).

### 2.8. Thiobarbituric Acid Reactive Substance (TBARS)

The TBARS levels of fish meat during refrigerator storage expressed as mg malondialdehyde (MDA)/kg were determined according to the method in Erol et al. [23] with little modification. The fish meat sample (10 ± 1 g) was mixed with 50 mL of 7.5% (w/v) trichloroacetic acid (TCA) and homogenized at 10,000 r/min for 1 min (T 25 easy clean digital, IKA, Guangzhou, China). The mixture was centrifuged at 10,000× *g* for 15 min at 4 °C and filtrated twice. Then, 5 mL of the filtrated solution was mixed with 5 mL of 0.02 M thiobarbituric acid (TBA) and kept in a boiling water bath for 30 min. The absorbance at 532 nm was measured, after cooling to room temperature.

### 2.9. Total Volatile Basic Nitrogen (TVB-N)

The TVB-N value was determined referring to the method of Cao et al. [24]. The fish meat was left to spread in 10 times of water for 30 min. The content of the TVB-N was determined using a Kjeldahl apparatus (KDy-9820, Beijing, China) and titrated by 0.01 M HCl.

### 2.10. Statistical Analysis

For data analysis, SPSS 24.0 software (SPSS Inc., Chicago, IL, USA) was employed. Origin 9.8.0 software was used for graphing. All experiments were carried out in triplicate; the results are reported as mean ± SEM. Duncan’s test was used to determine the significance (*p* < 0.05) for multiple comparisons, and *p*-values less than 0.05 were considered statistically significant.

## 3. Results and Discussion

### 3.1. Microbial Analysis

Within the same group, different lowercase superscripts indicate significant differences (*p* < 0.05); among different groups, different capital letter superscripts indicate significant differences (*p* < 0.05).

**Figure 2:**
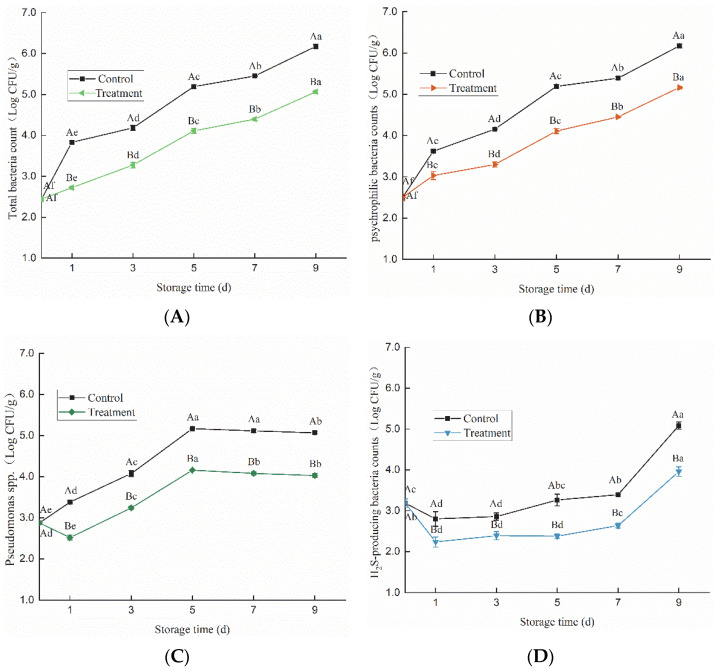

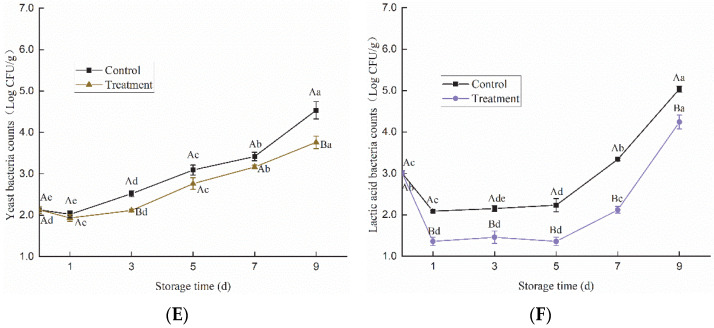
Changes in the number of microorganisms of *Trachinotus ovatus* during refrigerator storage (total bacteria count (**A**), *psychrophilic bacteria* (**B**), *Pseudomonas* spp. (**C**), H_2_S-producing bacteria (**D**), yeast (**E**), and lactic acid bacteria (**F**)).

As shown in Figure 2A,B, the total bacteria count and psychrophilic bacteria count in fish meat increased significantly (*p* < 0.05) during refrigeration storage, and their amounts in the HVACP treatment group were significantly lower (*p* < 0.05) than those of the control group. After nine days, the total bacteria count and psychrophilic bacteria count in the HVACP treatment group were 1.10 and 1.00 log CFU/g lower than those of the control group, respectively. *Pseudomonas* spp. in both the control and treatment groups increased quickly during the first five days of storage and reached 5.17 and 4.15 log CFU/g, respectively; then, they slightly decreased until day nine (Figure 2C). The amounts of H_2_S-producing bacteria decreased the first day and then increased gradually until day nine, as did the changes in yeast and lactic acid bacteria d (Figure 2D–F). The HVACP treatment effectively inhibited the growth of H_2_S-producing bacteria, yeast, and lactic acid bacteria during refrigerator storage. Compared with the control group, the number of colonies of H_2_S-producing bacteria, yeast, and lactic acid bacteria in the treatment groups was reduced by 1.12, 0.77, and 0.8 log CFU/g, after nine days of storage, respectively. This indicated that cold plasma treatment could effectively inhibit the growth of microbials in fish meat and maintain them at a low level during refrigerator storage.

A large number of RONS is generated during the HVACP treatment process; they can damage the cell wall and membrane to cause the leakage of cytoplasm [25], and parts of the RONS can move through the cell membrane to damage the nucleic acid, DNA, protein, and other important compositions inside the cell causing the death of cells [26]. Olatunde et al. [27] investigated the effects of DBD cold plasma treatment on the quality of Asian Sea Bass Slices packaged with 10% oxygen and 90% argon during storage, and they found that the microbial count in the treatment groups was still in the acceptable range after 12 d, while that of the control group was higher than the acceptable range after 6 d.

A high concentration of CO_2_ in the packaging could limit the growth of *Pseudomonas* spp., which are aerobic bacteria, and cause the bacteria to be at a disadvantaged condition in later bacterial competition. Li et al. [28] reported that MAP (CO_2_ 60%/5% O_2_/N_2_ 35%) combined with weakly acidic electrolyzed water significantly reduced the number of total colonies, H_2_S-producing bacteria, *Pseudomonas* spp., and Lactobacillus in Takifugu obscuru during storage, compared with both vacuum and air packaged samples. In this study, the number of microorganisms in the fish meat treated with HVACP did not exceed the limit of 10^7^ log CFU/g, which was still within the acceptable range [29], after nine days of storage. This indicated that HVACP treatment can inhibit the growth of microorganisms and is helpful to extend the shelf-life of fish meat.

### 3.2. Water Content

**Figure 3:**
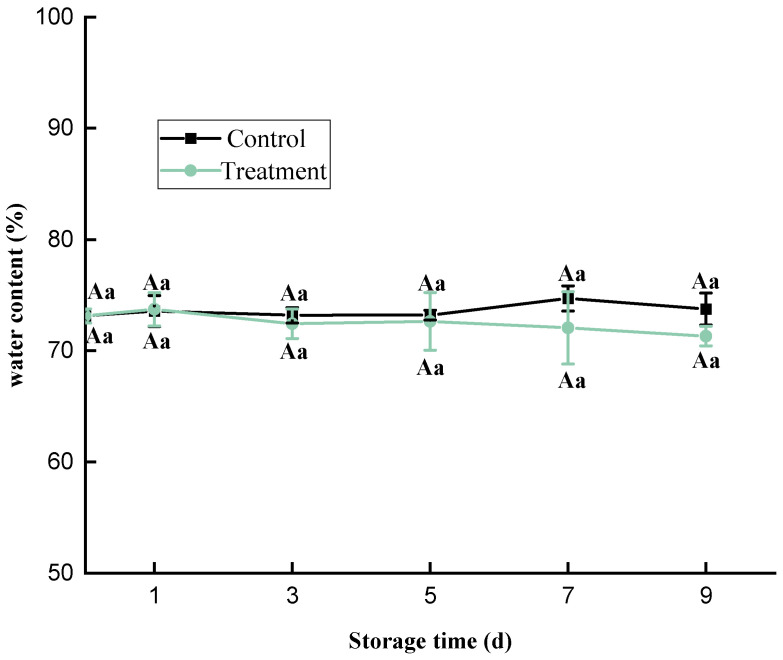
Changes in the water content of *Trachinotus ovatus* during refrigerator storage. Within the same group, different lowercase superscripts indicate significant differences (*p* < 0.05). Among different groups, different capital letter superscripts indicate significant differences (*p* < 0.05).

The water content is very important in fresh aquatic products; it can affect their freshness, taste, flavor, and texture. There was no significant change in the moisture content of the fish meat during the storage and no significant difference between the treatment and control groups (Figure 3), indicating that the HVACP treatment had no impact on the moisture content of fish. Similar results were reported in other food [30,31]. Pradeep et al. [17] found no significant difference in the water content of the flesh of saury between corona discharge plasma jet treatment and the control groups.

### 3.3. Color

As shown in Table 1, the L* value of treatment samples decreased gradually as the storage time extended, while there was no significant change in the L* value of the fish meat without treatment during refrigerator storage. With the same number of storage days, the L* value of the treated fish was significantly lower (*p* < 0.05) than that in the control group, the b* value was significantly higher (*p* < 0.05) than that in the control group, and there was no significant change in the a*.

These results indicated that the HVACP treatment had a negative influence on the lightness and yellowness of fish. The lipids’ oxidation accelerated by the ROS generated during the cold plasma treatment resulted in the damage of pigment proteins in the fish tissue, which affected the color of the fish meat; ROS can also cause oxidation reactions of myoglobin (Mb) and hemoglobin (Hb), namely color-forming substances, to result in a darker color of fish. Hydrogen peroxide, one main component of ROS, can react with myoglobin resulting in a yellowing tendency of fish during storage [32]. Moreover, the Maillard reaction could occur between free radicals containing carbonyl groups and amino acids [33], which causes an increase in yellowness. A similar result was found in cold plasma-treated herring fillets [34], the yellowness (b*) was increased during refrigerator storage. However, Albertos et al. [16], treated mackerel fillets for 1, 3, and 5 min with DBD cold plasma, found the L* value was slightly decreased, and found no difference in both a* and b* values after treatment. The different types of fish may have resulted in the different results between our study and the published papers.

### 3.4. Textural

**Figure 4:**
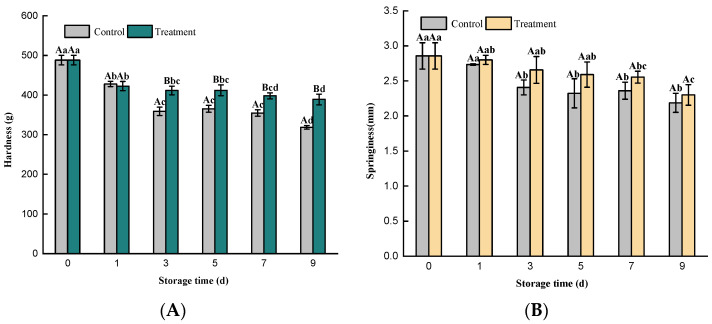

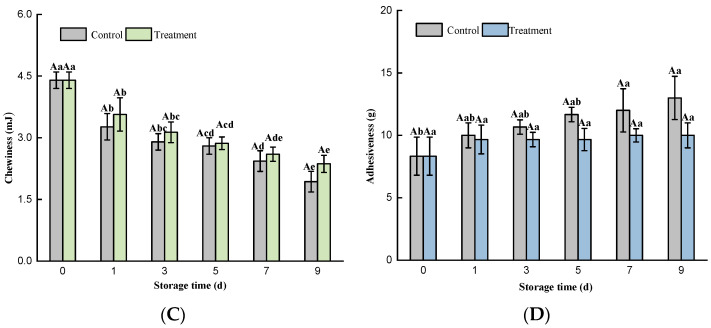
Changes in the hardness (**A**), springiness (**B**), chewiness (**C**), and adhesiveness (**D**) of *Trachinotus ovatus* during refrigeration. Within the same group, different lowercase superscripts indicate significant differences (*p* < 0.05); Different capital letters indicated significant differences (*p* < 0.05) between the treatment and control groups at same storage time.

The texture characteristics, as an important indicator for evaluating fish quality, can be affected by several factors, including post-slaughter treatment, the storage environment, the number of microorganisms, and the activity of endogenous enzymes [35]. As fresh material, the fish meat had relatively high initial hardness (Figure 4A, 0 d). After slaughtering, the activity of adenosine triphosphate (ATP) decreases, the concentrations of lactic acid and Ca^2+^ increases, actomyosin is formed through the cross-linking of myosin and actin, and rigor mortis causes a high hardness. Then, fish muscle protein is degraded by enzymes, and the meat changes from a rigor mortis state to an autolytic state, with a reduction in hardness. The hardness value of the treatment samples was always higher than that of the control group. HVACP treatment can accelerate the oxidation of proteins to limit the enzymatical degradation, which may be helpful to prevent a decrease in hardness. In addition, HVACP treatment can effectively inhibit the number of microorganisms in fish meat, which slows the degradation of protein and/or fat, and the muscle tissue softening resulting from the structural damage caused by microorganisms [36]. Choi et al. [37] used DBD to treat oysters and found that the length of time of the treatment had no significant effect on the oyster texture (hardness, springiness, cohesiveness, or resilience) compared to the control.

With the extension of the refrigeration time, the elasticity and chewiness of the samples showed a decreasing trend (Figure 4B,C). The HVACP treatment group was significantly lower (*p* < 0.05) than that of the control group. During the storage, the microorganisms in fish were inhibited effectively by RONS, which may reduce the activities of actomyosin and Ca^2+^, and the reduction in the salt-soluble protein in meat [38]. This is helpful to retain the elasticity and chewiness of fish. Manat et al. [39] treated Asian sea bass steak with plasma-activated water combined with ginger extract and found that the texture of fish meat decreased, which they believed might be caused by the destruction of the intact protein structure by microbial decomposition. The adhesiveness of the fish meat in the control group showed a significant upward trend during the refrigeration period (*p* < 0.05) (Figure 4D), and the adhesiveness in the HVACP treatment group showed a gentle trend. HVACP inhibited the growth and reproduction of microorganisms and reduced the amount of metabolites forming with nutrient decomposition on the surface by microorganisms. A similar result was reported in Xu et al. [40] who treated American herring with slightly acidic electrolyzed water (EOW) combined with chitosan (CH) and found that EOW+CH treatment effectively reduced the loss of firmness, elasticity, and chewiness during the refrigeration of fish meat, compared to EOW and CH alone. They believed that EOW+CH inhibited microbial growth during refrigeration in the American herring.

### 3.5. pH

**Figure 5:**
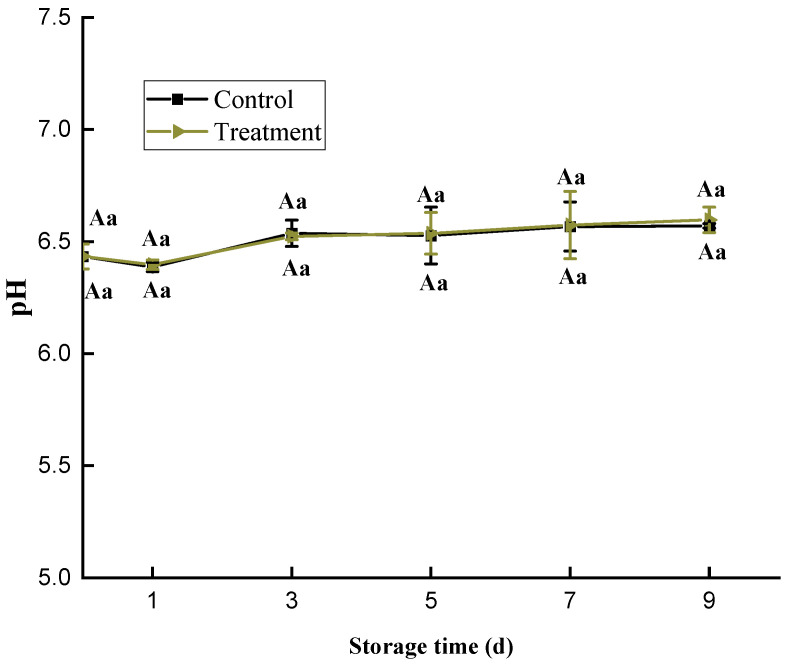
Changes in the pH of *Trachinotus ovatus* during refrigerator storage. Within the same group, different lowercase superscripts indicate significant differences (*p* < 0.05). Among different groups, different capital letter superscripts indicate significant differences (*p* < 0.05).

The pH value was decreased slightly and then increased during refrigerated storage, and there was no significant difference between the treatment group and the control group (Figure 5). This indicated that the HVACP treatment did not affect the pH of the samples. The pH of the fish decreased slightly in first day, because inorganic phosphate was released during ATP degradation at the early stage of fish death, and a large amount of lactic acid was generated through anaerobic glycolysis; all this increased the concentration of H^+^. In addition, the acidic free radicals generated in cold plasma may have reacted with the water in fish to form acidic components, which enhanced the decrease in pH. As the storage time extended, alkaline substances such as ammonia compounds and trimethylamine, were formed with the degradation of proteins caused by microorganisms. As a result, the pH began to increase after three days. Choi et al. [41] used DBD cold plasma to treat dried black mouth angler; they found the physicochemical properties of the dried black mouth angler were better than that of the control group, and there was no significant difference in color and pH.

### 3.6. TBARS

**Figure 6:**
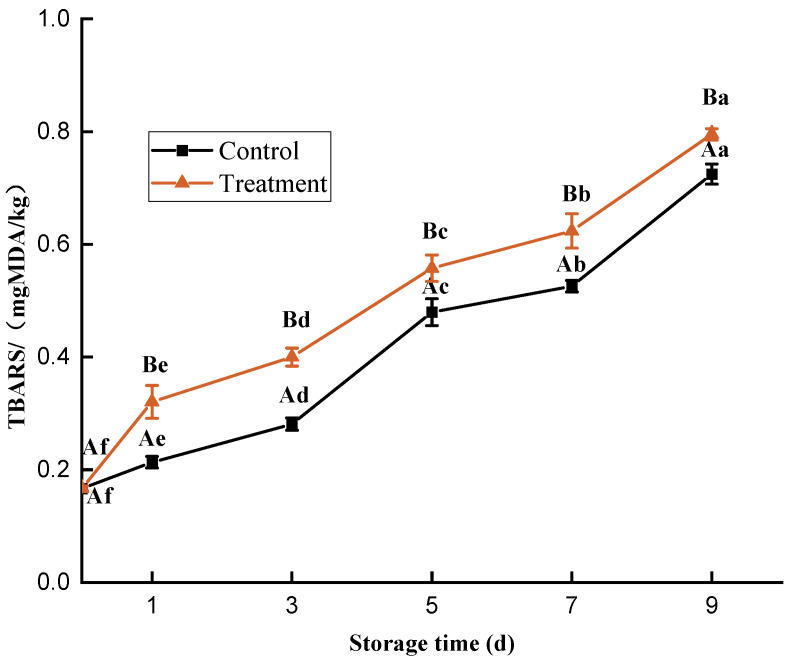
Changes in the TBA value of *Trachinotus ovatus* during refrigerator storage. Within the same group, different lowercase superscripts indicate significant differences (*p* < 0.05). Among different groups, different capital letter superscripts indicate significant differences (*p* < 0.05).

As shown in Figure 6, the TBARS value of the fish meat showed a statistically significant increase (*p* < 0.05) during refrigeration storage, the HVACP treatment group had a significantly higher (*p* < 0.05) value than that in the control group. The TBARS values in the treatment group was 0.79 mg/kg after nine days, which did not exceed the detection threshold of 2.0 mg/kg; if the TBARS value in fish exceeds 2.0 mg/kg, it indicates that the fish meat is corrupt and deteriorating, which has a negative impact on the quality and flavor of the fish meat [42].

The free radical species in ROS can induce and accelerate the lipid radical chain reaction, which leads to the increase in the TBARS value in meat [43]. In addition, fish meat is high in fat and rich in nutrients and is susceptible to microbial contamination, which leads to increased lipid oxidation in fish meat [7]. Kim et al. [44] treated bacon with plasma and found the degree of lipid oxidation of the treated group was higher than that of the control group during storage.

### 3.7. TVB-N

**Figure 7:**
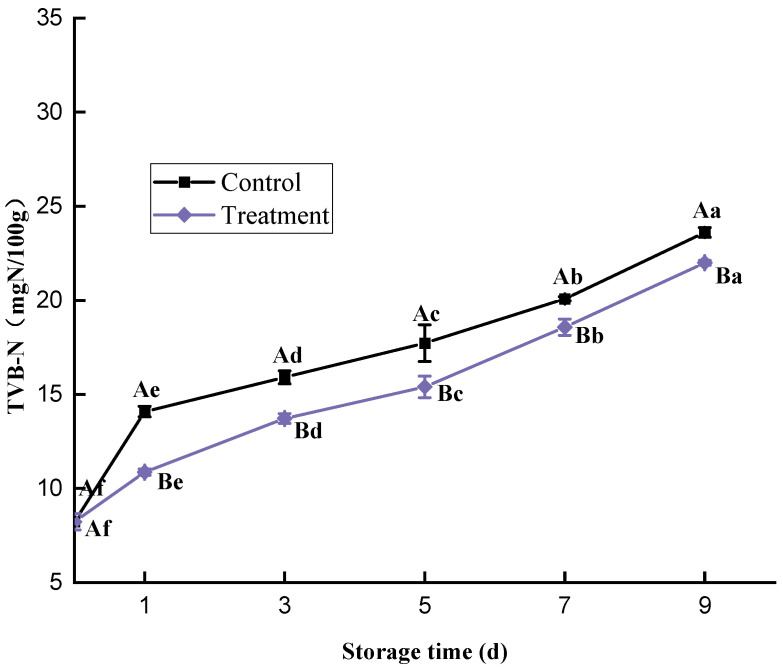
Changes in the TVB-N values of *Trachinotus ovatus* during refrigerator storage. Within the same group, different lowercase superscripts indicate significant differences (*p* < 0.05). Among different groups, different capital letter superscripts indicate significant differences (*p* < 0.05).

TVB-N, as an important indicator for judging the freshness of fish, is affected by the number of microorganisms and chemical changes caused by endogenous enzymes during storage [45]. The TVB-N value of fish meat was significantly increased (*p* < 0.05) during storage (Figure 7). The TVB-N value of the control group was always higher than that of treatment group. When refrigerated for nine days, the TVB-N value of the treatment group was 22.00 mg/100 g, still less than the limit standard of TVB-N in fresh sea fish (≤30 mg/100 g) [46]. The microorganisms increased as refrigeration time increased, they accelerate the decomposition of protein and non-protein substances in fish and produce alkaline metabolites such as amines to increase the TVB-N value [47]. In our study, HVACP treatment effectively inhibited the growth of microorganisms and controlled the increase in the TVB-N value caused by the decomposition of protein.

## 4. Conclusions

HVACP treatment can effectively inhibit the growth of microorganisms in *T. ovatus* during refrigerator storage. It also delayed the adverse effects of changes in texture, water content, pH, and the color of the fish. The treatment accelerated the lipid oxidation of fish to a certain extent but not exceed the critical value of fatty acid rancidity detection after nine days; the TVB-N content of the treated fish meat also met the standard for fresh marine fish after nine days.

In our study, the refrigerator shelf-life of *T. ovatus* after HVACP treatment was at least nine days. HVACP treatment slowed the deterioration of the physical and chemical indicators of fish meat during refrigeration. Therefore, HVACP is a useful technology for extending the shelf-life of *T. ovatus* and a potential method to maintain the quality and prolong the shelf-life of other aquatic products in the future.

## Figures and Tables

**Table 1:** Changes in the color of *Trachinotus ovatus* during refrigerator storage.

Storage Time (d)	Group	L*	a*	b*
0	control	56.50 ± 0.32 ^Aa^	1.77 ± 0.03 ^Aa^	6.03 ± 0.13 ^Aab^
	treatment	56.50 ± 0.32 ^Aa^	1.77 ± 0.03 ^Aa^	6.03 ± 0.13 ^Aa^
1	control	55.87 ± 0.09 ^Ab^	1.90 ± 0.06 ^Aa^	5.77 ± 0.12 ^Aa^
	treatment	55.40 ± 0.4 ^Aa^	1.73 ± 0.05 ^Aa^	5.90 ± 0.11 ^Ab^
3	control	55.53 ± 0.07 ^Ab^	1.93 ± 0.03 ^Aa^	6.07 ± 0.15 ^Aa^
	treatment	54.70 ± 1.09 ^Aab^	1.83 ± 0.06 ^Aa^	6.27 ± 0.19 ^Aab^
5	control	55.40 ± 0.08 ^Ab^	1.83 ± 0.08 ^Aa^	5.93 ± 0.19 ^Aa^
	treatment	54.83 ± 0.35 ^Aab^	1.80 ± 0.05 ^Aa^	6.47 ± 0.18 ^Aa^
7	control	55.30 ± 0.06 ^Ab^	1.90 ± 0.05 ^Aa^	5.90 ± 0.21 ^Aa^
	treatment	53.40 ± 0.27 ^Bbc^	1.77 ± 0.07 ^Aa^	6.23 ± 0.18 ^Aab^
9	control	55.70 ± 0.07 ^Ab^	1.87 ± 0.09 ^Aa^	5.73 ± 0.15 ^Aa^
	treatment	52.70 ± 0.55 ^Bc^	1.73 ± 0.03 ^Aa^	6.37 ± 0.12 ^Bab^

Note: different lowercase superscripts indicate significant differences (*p* < 0.05) in the same group between different days; different capital letter superscripts indicate significant differences (*p* < 0.05) in different groups in same day.

## Data Availability

Data are contained within the article.
